# Fold change and p-value cutoffs significantly alter microarray interpretations

**DOI:** 10.1186/1471-2105-13-S2-S11

**Published:** 2012-03-13

**Authors:** Mark R Dalman, Anthony Deeter, Gayathri Nimishakavi, Zhong-Hui Duan

**Affiliations:** 1 Department of Biology and program in Integrative Bioscience, University of Akron, Akron, OH, USA; 2 Department of Computer Science, University of Akron, OH, USA

## Abstract

**Background:**

As context is important to gene expression, so is the preprocessing of microarray to transcriptomics. Microarray data suffers from several normalization and significance problems. Arbitrary fold change (FC) cut-offs of >2 and significance p-values of <0.02 lead data collection to look only at genes which vary wildly amongst other genes. Therefore, questions arise as to whether the biology or the statistical cutoff are more important within the interpretation. In this paper, we reanalyzed a zebrafish (*D. rerio*) microarray data set using GeneSpring and different differential gene expression cut-offs and found the data interpretation was drastically different. Furthermore, despite the advances in microarray technology, the array captures a large portion of genes known but yet still leaving large voids in the number of genes assayed, such as leptin a pleiotropic hormone directly related to hypoxia-induced angiogenesis.

**Results:**

The data strongly suggests that the number of differentially expressed genes is more up-regulated than down-regulated, with many genes indicating conserved signalling to previously known functions. Recapitulated data from Marques et al. (2008) was similar but surprisingly different with some genes showing unexpected signalling which may be a product of tissue (heart) or that the intended response was transient.

**Conclusions:**

Our analyses suggest that based on the chosen statistical or fold change cut-off; microarray analysis can provide essentially more than one answer, implying data interpretation as more of an art than a science, with follow up gene expression studies a must. Furthermore, gene chip annotation and development needs to maintain pace with not only new genomes being sequenced but also novel genes that are crucial to the overall gene chips interpretation.

## Background

As more and more genomes are sequenced and annotated, the capacity to accurately and efficiently catalog the gene expression profiles of these organisms is becoming ever more apparent [1]. With techniques such as in situ hybridization, QRT-PCR, and more recently absolute quantitation being used to assess gene expression, there are still lingering issues of humble throughput and lack of massive parallel comparisons. Array technology has improved these conditions yet problems of standardizing statistical analyses are lacking, along with observed differences when comparing microarray platforms [2], though others have found significant reproducibility [3].

Oligonucleotide arrays, for example, prove useful in not requiring cDNA library production [4], whilst cDNA microarray proves useful in cases of non-model organism and even been used to identify heterologous genes across multiple species [5]. Even so, the current microarray platforms are still several years behind the current state of knowledge of many organisms genome. Furthermore, classifying a differentially regulated gene is a problem of both array types with research even suggesting it should be dealt with in a tiered approach [6].

To assess the power of analysis, there are many different ways when it comes to expression data [7-9]. The use of t-tests, ANOVAs, Gene Ontology (GO) annotation, p-value cutoffs, Bonferroni corrections, array normalization, Fishers exact test, and fold change cut offs all lead towards a reduction in gene expression data which may inadvertently reduce or increase the power of analysis.

We obtained a data set from a recently published paper and reanalyzed the raw data using multiple different approaches. From this data analysis, we hypothesized that by changing the significance level as well as the fold change cut off, more than one interpretation of the data can be obtained. Subsequently, there is only one microarray study on heart tissue response to hypoxia published, implying a low value for replication and the possibility of perpetuation of error and to the accumulation of unreliable explanations and unverified findings. Essentially the novelty of this study in light of Marques et al. (2008) is for future studies to unravel which significance criteria is relevant, biological or statistical.

## Results and discussion

**Figure 1:**
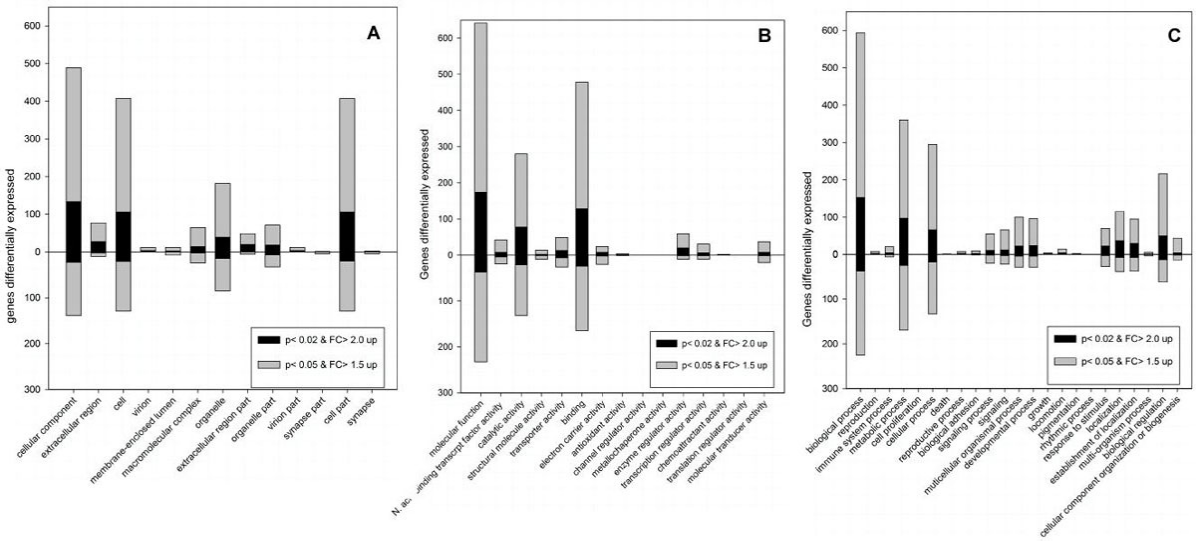
**Differentially regulated genes for GO Annotation categories**. A. Cellular component B. Molecular function C. Biological Function. Black shaded blocks are the intersection of genes with p-values ≤ 0.02 and fold change cutoff of ≥2.0. Gray shaded blocks are the intersection of genes with p-values ≤ 0.05 and fold change cutoff of ≥1.5. The genes found in the dark shaded blocks are also included in the number of genes in the gray shaded block. Categories are directly taken from the second level of GO annotation from GeneSpring.

Microarray technology has proven beneficial to directly identifying co-regulated genes, pathways, and systems allowing for a more informed snapshot of the transcriptome. As such, our results indicate that changes in the significance level of differential expressed gene products along with the fold change cut-offs can give very different results that imply different signaling pathways and functions involved (Figure 1). As T-tests have been widely used to identify deviation from the mean, large sampling sizes (~15,000 genes assayed) can influence the number of false positives and may infer little if anything about the biology [9,10]. Fold change on the other hand lends itself to a more biologically meaningful assessment yet still encounters problems with identifying what is significant to the organism. Therefore using both criteria may help but not fix the problem of microarray analysis.

Within this study, the contributions of each differentially expressed gene criteria were evaluated. As indicated before, the criteria suggest different biological meaning. The number of significant genes were overwhelming at a p ≤ 0.05 and even upon increasing to a p ≤ 0.02 level, the data is reduced almost in half yet still remains massive for understanding the biological response to hypoxia (Supplemental 1, Table 1). Fold change suggests more meaningful insight to the organism throughout development and into adulthood [11] with 1.5 proving to be a better eliminator of background noise as there were fewer genes left after making a fold change cutoff of ≥1.5 as compared to using significance cutoffs (Supplemental 1, Table 1). As the fold change level increases to that of ≥2, the number of genes significantly decreases. This suggests that biologically, less genes change drastically and that the significant difference observed at p ≤ 0.05 and 0.02 are related to a possible whole animal response to treatment. To understand the change and ultimately the importance in interpretation of genes influenced by chronic constant hypoxia, an intersection of ≥1.5 fold change and p ≤ 0.05 was compared to ≥2 fold change and p ≤ 0.02.

The data obtained from our analysis indicates that there were more up-regulated genes as compared to down regulated, implying that even in a state of stress the organism is actively adjusting its transcriptome and, more specifically, the transcriptome of the heart (Figures 1, supplemental 1).

Using the most stringent criteria (intersection of p ≤ 0.02 and ≥2 fold change), there were several genes that were up-regulated and observed in both intersection groups (Figures 1, supplemental 1). Hypoxia inducible factor 1 was found to be up-regulated and is known to be expressed in response to hypoxic conditions [12]. Interestingly, chemokine (C-X-C motif) ligand 12a stromal cell derived factor 1 was only observed under ≥1.5 fold change and p ≤ 0.05 conditions. CXCL12 is involved in directing hematopoietic cells and angiogenesis [13]. By omitting this chemokine from the biological interpretation, clinical researchers may overlook a corollary between tumor progression (which requires oxygen) and ischemia, a state of desperate need for oxygen and nutrients. Other genes such as bone morphogenetic protein 2a and insulin-like growth factor binding protein 1a were observed in each selection criteria indicating they are significant and change more than two fold in response to chronic constant hypoxia. IGFBP-1A has been known to be involved in regulating IGF and insulin pathways which can be linked to cell proliferation and protection against cell death along with BMP-2 involved with bone development [14,15].

Those genes down- regulated at the most stringent selection criteria show similar reductions to those observed by Marques et al (Figure 1) [16]. Metabolically related genes, such as acyl coenzyme A dehydrogenase, pyruvate dehydrogenase kinase, SOCS3, and creatine kinase indicate that a shift to oxygen independent metabolism is occurring (Additional file). These genes are involved in fatty acid oxidation, pyruvate oxidation, cytokine signaling disruption, and the rapid shuttling of ATP sources, respectively. Interestingly, changes both at a metabolic level as well as at a synaptic level were observed as synaptotagmin, uncoupling protein 2, and ATPase, Ca^2+ ^transporting, cardiac muscle, fast-twitch 1 like gene were found to be significantly down regulated indicating that mitochondrial membrane leak was reduced and calcium initiated cellular signaling may be attenuated in cardiac tissue (Figure 1, Additional file).

However, some genes not identified under the most stringent criteria and counter to ischemia/ reperfusion, are superoxide dismutase, heat shock protein 90, and lactate dehydrogenase D. This suggests the response to chronic constant hypoxia may be tissue specific as these genes should be up-regulated under hypoxic conditions [17]. SOD has been found to be crucial in the breakdown of reactive oxygen species while HSP are used for stabilizing proteins under stressful conditions [18]. Other genes such as programmed cell death 8 (apoptosis-inducing factor) and RAS association (RalGDS/AF-6) domain family 8 were down regulated and differentially expressed but were not found under most stringent statistical criteria. Both of these genes are involved in cellular processes important to the survival of the cell or its interaction with other cells, respectively. As zebrafish have developed ways to respond and survive under hypoxic conditions, AIF-8 and RAS may bring insight into cellular mechanisms responsible for cell survivability and cell rigidity [19]. Thus understanding the integrative response to chronic constant hypoxia is beginning to look more and more like the response observed in tumorigenesis, which may be overlooked based on selection criteria of genes.

Using fold enrichment analysis and fishers exact tests of all sets in comparison to originally published, we found that though our most stringent criteria was similar to Marques et al [16], we had distinct changes in GO annotation groups involved in microtubule activity, hydrolase activity, nucleic acid binding and carbohydrate binding (Additional file). Interestingly, reducing the criteria down to 1.5 FC and p of 0.05, several different GO gro*u*ps were found to be significant especially those involved in structural integrity, ribosome structure, and transcription regulation and factors, suggesting these may not vary widely in expression but that there are more significant genes thus pointing towards the problems of microarray analysis (Additional file). At one spectrum, novelty in research is revered whilst on the other hand replication and accuracy to natural phenomena may result in skewed explanations. We humbly feel the reanalysis of microarray data is necessary along with follow up gene expression studies to accurately explain biological phenomena.

## Conclusions

Our results point to the dangers of statistical selection criteria as well as shed light on genes crucial to understanding chronic constant hypoxia. As more genes are sequenced, microarray platform expanded, along with understanding the role of splice variants in zebrafish (*D. rerio*), a larger and more dynamic picture of the transcriptome can be gathered. In sum, we demonstrate how fold change and statistical cut-offs modulate the outcome of microarray data. Future, studies should tie together previously unanalyzed genes such as leptin to microarray data in response to constant hypoxia in adult zebrafish. As the current microarray platform does not include them, problems still exist at the molecular level, influencing our understanding of the physiological and ultimately behavioural and ecological roles of these organisms.

## Methods

In a previously published paper in which adult zebrafish heart tissue was assayed in response to chronic hypoxia [16], microarray files were downloaded from NCBI Gene Expression Omnibus (http://www.ncbi.nlm.nih.gov/geo) (including accession numbers GSM112796 and GSM112798 through 806). Raw files were imported into GeneSpring GX Version 11.0 (Silicon Genetics, Redwood City, CA) and intensities normalized using MicroArray Suite 5 method [20]. Due to small sample size, equal variance across data could not be assumed and data was analyzed using an unpaired TTest with unequal variance with no statistical corrections [21]. Resultant data was pooled into four groups (Table 1).

## Competing interests

The authors declare that they have no competing interests.

## Authors' contributions

MRD prepared the manuscript. AD utilized the GeneSpring software, designed and implemented the filtering software, and compiled the data. MRD and AD analyzed the data. Manuscript was reviewed by MRD, AD, and ZD.

## Supplementary Material

Additional file 1 **Comparative Fishers exact test and fold enrichment for significant GO groups within the zebrafish total gene array**. Viewable in excel. Click here for file
